# Discovery of a new molecule inducing melanoma cell death: dual AMPK/MELK targeting for novel melanoma therapies

**DOI:** 10.1038/s41419-020-03344-6

**Published:** 2021-01-11

**Authors:** Emilie Jaune, Elisa Cavazza, Cyril Ronco, Oleksandr Grytsai, Patricia Abbe, Nedra Tekaya, Marwa Zerhouni, Guillaume Beranger, Lisa Kaminski, Frédéric Bost, Maeva Gesson, Meri Tulic, Paul Hofman, Robert Ballotti, Thierry Passeron, Thomas Botton, Rachid Benhida, Stéphane Rocchi

**Affiliations:** 1 INSERM, U1065, Equipe 12, Centre Méditerranéen de Médecine Moléculaire (C3M), Bâtiment ARCHIMED, 151 route de saint Antoine de Ginestière, 06204 Nice cedex 3, France; 2 Equipe Labellisée Fondation pour la Recherche Médicale (FRM) 2020, Paris, France; 3 Université Cote d’Azur, Nice, France; 4 Institut de Chimie de Nice UMR-CNRS 7272, Université Nice Sophia Antipolis, Parc Valrose, 06108 Nice Cedex 2, France; 5 INSERM, U1065, Equipe 5, Centre Méditerranéen de Médecine Moléculaire (C3M), Bâtiment ARCHIMED, 151 route de saint Antoine de Ginestière, 06204 Nice cedex 3, France; 6 UFR de Médecine, Université de Nice Sophia Antipolis, 06000 Nice, France; 7 Institute of Research on Cancer and Ageing of Nice (IRCAN), INSERM U1081, CNRS UMR7284, Nice, 06107 France; 8 Laboratoire de pathologie clinique et expérimentale et Hospital-related biobank (BB-0033-00025), Hôpital Pasteur, 06002 Nice, France; 9 INSERM, U1065, Equipe 1, Centre méditerranéen de Médecine Moléculaire (C3M), Bâtiment ARCHIMED, 151 route de saint Antoine de Ginestière, 06204 Nice cedex 3, France; 10 Service de Dermatologie, Hôpital Archet II, CHU, 06204 Nice, France

**Keywords:** Melanoma, Cell death

## Abstract

In the search of biguanide-derived molecules against melanoma, we have discovered and developed a series of bioactive products and identified the promising new compound CRO15. This molecule exerted anti-melanoma effects on cells lines and cells isolated from patients including the ones derived from tumors resistant to BRAF inhibitors. Moreover, CRO15 was able to decrease viability of cells lines from a broad range of cancer types. This compound acts by two distinct mechanisms. First by activating the AMPK pathway induced by a mitochondrial disorder. Second by inhibition of MELK kinase activity, which induces cell cycle arrest and activation of DNA damage repair pathways by p53 and REDD1 activation. All of these mechanisms activate autophagic and apoptotic processes resulting in melanoma cell death. The strong efficacy of CRO15 to reduce the growth of melanoma xenograft sensitive or resistant to BRAF inhibitors opens interesting perspective.

## Introduction

Melanoma is the most aggressive skin cancer and possesses a strong invasive capability that allows the development of metastasis principally in the lymph nodes, liver, lungs, and central nervous system. Metastatic melanoma is one of the deadliest cancers because of its ability to develop resistance to current therapies.

For 15 years, targeted therapies against BRAF (mutated in 50–60% of primary melanoma) or MEK protein has been developed, and some of them are commercialized, including BRAF inhibitors such as vemurafenib (or PLX4032), dabrafenib and encorafenib, as well as MEK inhibitors such as selumetinib, trametinib, cobimetinib or binimetinib^1^. The results with combination between BRAF and MEK inhibitors seem promising with an increase in the median progression-free survival (9.9 months for combination versus 6.2 months for BRAF inhibitors alone) and shrinkage of the tumor^2,3^. However, after a few weeks of treatment, patients develop strong resistance to therapies, allowing metastasis growth and relapse^4–6^.

Melanoma cells have the capacity to escape the immune response. This observation led to the development of immune checkpoint inhibitors allowing the reactivation of the immune system to kill melanoma cells^7,8^. Currently, two different types of immunotherapies are available in the form of blocking antibodies: Ipilimumab (anti-CTLA-4) and Nivolumab or Pembrolizumab (anti-PD-1). Ipilimumab targets the CTLA-4 receptor of CD4+ T cells allowing their activation. This treatment increases survival rate in patients, but only 15% respond to this treatment^9^. PD-1 is also expressed on T cells and its expression inhibits T cell activation. Its target PDL-1 is widely found in melanoma cells. Anti-PD-1 treatment elicits a response in approximately 30% of patients^10^. Even if these responses are objective and long-lasting, approximately 65–70% of patients do not respond to this treatment. More recently, the result of phase III trial using combination of ipilimumab and nivolumab demonstrated an increase of efficacy compared with ipilimumab alone with the detriment of higher toxicity^11^. Indeed, grade 3/4 toxicity rate were observed in 55% cases for combination therapy. Thus, identification of new anti-melanoma compounds is essential to developing new therapies.

In this perspective, we previously used a structure/function approach that led to the characterization of the anti-melanoma properties of Thiazolbenzenesulfonamide (TZB). We thus identified HA15 as the lead compound of this family, able trigger melanoma cell death by induction of a strong endoplasmic reticulum stress^12^.

Here, we use a similar approach to improve the biological activity of biguanides on melanoma.

Metformin is the most commonly used biguanide in diabetic treatment. It assumes little side effects and is well tolerated. A number of studies have shown that metformin induces cell death by apoptosis in numerous types of cancer, including melanoma^13^. We have shown that metformin is responsible for cell cycle arrest of melanoma cells in G1–G0 after 24 h of treatment^14–16^. This cell cycle arrest induces AMPK pathway activation, which activates autophagy and apoptosis signaling cascades, leading to melanoma cell death. In vivo, we have demonstrated that metformin decreases tumor growth in a xenograft mouse model^14^. Furthermore, we have shown that metformin decreases the invasive capacity of melanoma cells in an AMPK/p53 dependent manner without affecting their cell migration ability^16^. These results were confirmed by the observation that metformin strongly decrease in melanoma pulmonary metastasis formation in C57BL/6 mice. These results prompted us to perform a multicentric clinical trial aiming to test the efficacy of metformin as monotherapy for melanoma treatment. Unfortunately, very few patients responded to this treatment^17^. It is worth noting that metformin has low activity (IC_50_ in the millimolar range) and low oral bioavailability requiring therefore high daily dose for treatment of cancer. Moreover, taking into account the small molecular size of metformin, its pleiotropic effects and mechanism of action remain unclear and controversial.

As a result, we started a medicinal chemistry program aimed at developing new metformin-derived molecules with improved potency and suitable pharmacological profile to obtain a better response in patients. After an initial screening, an extensive structure-activity-based hit-to-lead optimization led us to identify a new potential candidate called CRO15.

## Results

### CRO15 exerts deleterious effects on melanoma cell viability

**Fig. 1:**
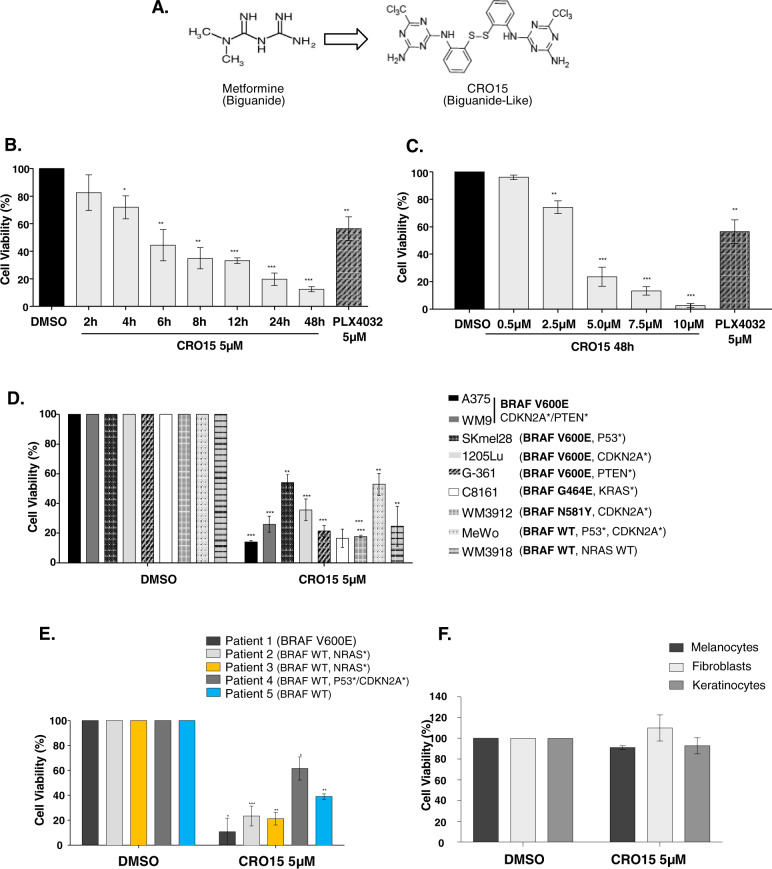
CRO15 exerts a deleterious effect on melanoma cell viability. **A** Chemical structure of metformin and CRO15. **B**–**F** Cell viability assay using trypan blue exclusion method on **B**. A375 melanoma cells treated with 5 μM CRO15 at different times. **C** A375 melanoma cells were treated with the indicated concentrations of CRO15 for 48 h. **D** Melanoma cell lines of various genotypes treated with 5 μM CRO15 for 48 h. **E** Human melanoma cells freshly isolated from tumors treated for 48 h with 5 μM CRO15. **F** Primary human melanocytes, keratinocytes and fibroblasts treated 48 h with 5 μM CRO15. For **B** to **F**, results are expressed as percentages of control and data given as the means ± SEM of three independent experiments performed in triplicate. *p < 0.05; **p < 0.01; ***p < 0.001.

After extensive screening of new molecules derived from metformin, based on structure/activity characteristics, a lead compound, called CRO15, with an original structure was discovered. This molecule, described in Fig. 1A, shows a biguanide cyclized form, with an additional aromatic ring compared to metformin.

The effect of CRO15 on melanoma cells viability was determined using a blue trypan exclusion assay. Time course experiments showed that after 4 h of treatment with 5 μM CRO15, melanoma cell viability decreased in comparison to the DMSO control condition (Fig. 1B). These experiments were performed in A375 melanoma cells, which are sensitive to BRAF inhibitors. After 48 h of treatment, only 15% of melanoma cells were still alive.

Figure 1C shows a dose-dependent response after 48 h of treatment of A375 melanoma cells with CRO15. The 50% inhibitory concentration (IC_50_) of this molecule was identified to be between 2.5 and 5 μM. Our previous results obtained on the same cell line demonstrated that metformin was inducing cell death after 96 h of treatment at 5 and 10 mM^14^. The effects of CRO15 were confirmed on other melanoma cell lines with various mutational statuses (Fig. 1D). We observed a large decrease in melanoma cell viability after 48 h, independent of their mutations. The anti-melanoma effects of CRO15 were also confirmed in a more physiological model of melanoma cell cultures freshly isolated from patient tumors with BRAF mutation (Patient 1), NRAS mutation (Patients 2 and 3), PTEN and p53 mutations (Patient 4) and with no know mutation (Patient 5). Importantly, we no toxicity of CRO15 (5 μM) was observed on normal human cells, such as melanocytes, fibroblasts and keratinocytes, at 48 h of treatment (Fig. 1F).

### CRO15 induces a mitochondrial disorder and activates AMPK pathway

To understand the signaling pathway induced by CRO15, we first examined metabolic alterations and AMPK pathway activation. Indeed, it is known that metformin can induce the AMPK (*AMP-activated* protein kinase) pathway by inhibition of the complex I of the respiratory chain of mitochondria.

**Fig. 2:**
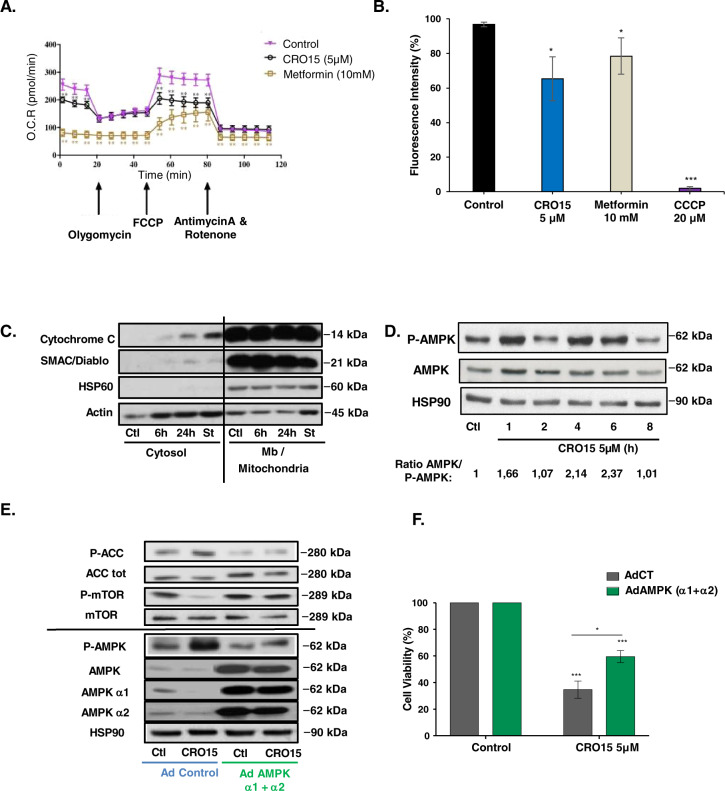
CRO15 induces a mitochondrial disorder and activates AMPK pathway. **A** Measurement of mitochondrial function in A375 melanoma cells with a Seahorse XF Cell Mito stress test after 6 h of treatment with 5 μM CRO15, 10 mM metformin or DMSO used as control. At 20 min, cells are treated with oligomycin, an inhibitor of complex V of respiratory chain; at 50 min, with FCCP, which disrupts the mitochondrial membrane potential; and at 80 min, with Rotenone and antimycin A, inhibitors of complex I and III, respectively. **B** A375 cells were treated with 5 μM CRO15 (6H), 10 mM metformin (6H) for or 20 μM CCCP (20 min). Measurement of mitochondrial potential membrane was performed by flow cytometry with a TMRE probe. **C** Lysates from A375 melanoma cells exposed for the indicated durations to CRO15 were analyzed by western blotting using the indicated antibodies after cell fractionation. One representative experiment of three is shown. **D** Lysates from A375 cells treated with 5 μM CRO15 for the indicated durations were analyzed by western blotting using the indicated antibodies. One representative experiment of three is shown. **E** Lysates form A375 cells transduced with dominant negative AMPK subunits α1 and α2 for 24 h and treated with 5 μM CRO15 were analyzed by western blotting using the indicated antibodies. One representative experiment of three is shown. **F** Quantification of cell viability from **E** using trypan blue exclusion method. Results are expressed as percentages of DMSO control and data given as the means ± SEM of three independent experiments performed in triplicate. *p < 0.05; **p < 0.01; ***p < 0.001.

To determine whether CRO15 has the same effects as metformin, we first studied the mitochondrial potential of melanoma cells after drug treatment (6 h) by Seahorse experiments (Fig. 2A). We observed a significant reduction in basal mitochondrial respiration in response to CRO15, which was not as great as that seen with metformin. Interestingly, the results showed a problem in restoration of maximal respiration after FCCP injection. This experiment demonstrates that although CRO15 does not have exactly the same efficiency as metformin on mitochondrial respiration, it induces significant dysregulations. To confirm the effects of CRO15 on mitochondrial metabolism, we measured mitochondrial transmembrane potential in melanoma cells (Fig. 2B). We used TMRE, a cell-permanent dye that readily accumulates in active mitochondria. Depolarized or inactive mitochondria have decreased membrane potential and fail to sequester TMRE. We observed a depolarization of the mitochondria in response to metformin (10 mM) but also CRO15 treatment (5 μM). This result was confirmed by a decrease in ATP production after CRO15 treatment (Supplemental Fig. 1A) and a release of cytochrome C and SMAC/Diablo in the cytoplasm after 6 and 24 h of treatment with CRO15 (Fig. 2C).

Dysregulation of mitochondria implicated activation of signaling pathways such as the AMPK pathway. Indeed, a decrease in ATP/AMP ratio is responsible for activation by phosphorylation of AMPK. Thus, treatment with CRO15 induced a time-dependent phosphorylation of AMPK on multiple melanoma cell lines (Fig. 2D and Supplemental Fig. 1B) but had minimal effects on melanocytes and fibroblasts consistent with its lack of toxicity normal cells (Supplemental Fig. 1C).

AMPK kinase can act on many effectors. For example, AMPK activation can induce the phosphorylation of ACC (*Acetyl-CoA carboxylase)* and the dephosphorylation of mTOR (*mammalian target of rapamycin*) and thus inactivates their activities and stop diverse metabolisms activities. To confirm the involvement of the AMPK pathway in the mechanism of action of CRO15, we infected A375 cells with adenoviruses expressing dominant negative forms of AMPK α1 and α2, the two catalytic subunits of AMPK kinase. These adenoviruses, by expression of dominant negative forms of AMPK, shut down activation of endogenous AMPK (Fig. 2E) and patially restored cell viability of melanoma cells treated with CRO15 (5 μM) for 24 h (Fig. 2F). These results demonstrate that the AMPK pathway plays a role in CRO15-induced decrease in melanoma cell viability.

### CRO15 inhibits MELK kinase activity

**Fig. 3:**
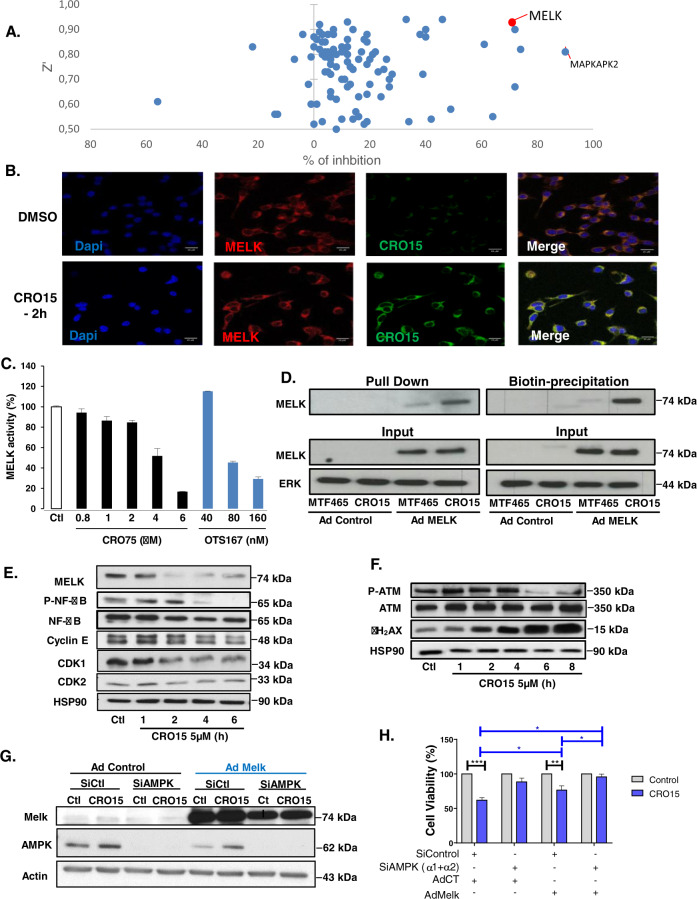
CRO15 inhibits MELK kinase activity. **A** Scatter plot representing the percentage of kinase inhibition and their Z′ factor measured during two independent in vitro kinome assay with 1 μM CRO15. **B** Immunofluorescence pictures of CT26 cancer cells treated with 5 μM CRO15 for 2 h. MELK was labeled with a specific antibody (in red), CRO15 was coupled to a fluorescent tag (in green) and nuclei were stained with DAPI (in blue). **C** In vitro test of kinase activity using recombinant MELK protein treated with CRO15 with the MELK inhibitor OTS167. **D** A375 cells were infected with MELK adenovirus for 24 h. For the pull down assay, cells lysates were incubated overnight with 100 μM Biotin-CRO15 or its inactive analog MTF465. For Biotin precipitation, cells were treated for 2 h with 20 μM Biotin-CRO15 or MTF465 before lysis and western blot analysis with the indicated antibodies. One representative experiment of three is shown. **E** and **F** A375 cells were treated with 5 μM CRO15 with the indicated durations, and lysates were analyzed by western blotting using the indicated antibodies. One representative experiment of three is shown. **G** A375 cells were transfected with siRNA directed against AMPK α1 and AMPK α2 (20 nM each) or 40 nM of control siRNA. Twenty-four hours later, cells were infected with a MELK adenovirus or a control adenovirus before treatment with 5 µM of CRO15. After 24H, viable cells were counted using the trypan blue dye exclusion method (*n* = 3) (right panel). In parallel, cell lysates were analyzed by western blotting with the indicated antibodies (left panel). One representative experiment of three is shown.

To discover other pathways implicated in CRO15 mechanisms, we performed a kinome assay in comparison to metformin in which more than 120 kinases involved in cancer were analyzed (Table 1). This in vitro experiment allowed us to observe the percentage of direct modulation of different kinase activities induced by CRO15 in function of Z′ factor (Fig. 3A). We showed a strong inhibition of activity of MAPKAPK2 (inhibition: 90%, Z′: 0.82) and MELK kinase (inhibition: 72%, Z′: 0.90). Our results regarding MAPKAPK2, also known as MAPK2, indicate that this kinase is not involved in AMPK-independent effects of CRO15 in intact cells as its downstream effector HSP27 is not modulated (Supplemental Fig. 2A). Indeed, MAPK2 is also directly regulated by p38 MAPK protein, which, is activated under CRO15 treatment.

**Table 1:** Results of in vitro Kinome assay in response to CRO15 (1 μM) or metformin (1 mM) (*n* = 2).

Kinase			CRO15 1 µM	Metformin 1 mM
MAPKAPK2	Activity	Km app	90	−2
AKT2 (PKB beta)	Activity	100	74	3
GSK3B (GSK3 beta)	Activity	Km app	72	2
MELK	Activity	Km app	72	2
JAK2	Activity	Km app	64	−1
MAPK8 (JNK1)	Binding		64	−5
FER	Activity	Km app	61	7
SRC	Activity	Km app	49	22
ABL1 T315I	Activity	Km app	46	0
CHEK1 (CHK1)	Activity	Km app	42	6
AKT1 (PKB alpha)	Activity	Km app	40	0
YES1	Activity	Km app	40	7
AURKA (Aurora A)	Activity	Km app	39	4
WEE1	Binding		39	4
GSK3A (GSK3 alpha)	Activity	Km app	38	5
MAPK9 (JNK2)	Binding		36	−3
EPHA7	Binding		35	−7
BRAF V599E	Activity	100	34	4
CDK5/p35	Activity	Km app	33	4
AMPK (A1/B2/G3)	Activity	Km app	32	1
CHEK2 (CHK2)	Activity	Km app	28	6
MAP3K14 (NIK)	Binding		28	0
ABL1	Activity	Km app	27	3
MAPK10 (JNK3)	Binding		27	0
MST1R (RON)	Activity	Km app	27	−2
BLK	Activity	Km app	26	0
MAPK7 (ERK5)	Activity	Km app	25	4
MST4	Activity	Km app	24	15
AMPK (A1/B2/G2)	Activity	Km app	23	7
FGFR1	Activity	Km app	23	−12
RPS6KB1 (p70S6K)	Activity	Km app	22	1
ERN1	Binding		21	1
KDR (VEGFR2)	Activity	Km app	21	3
AMPK (A1/B1/G2)	Binding		20	2
MET (cMet)	Activity	Km app	20	−4
PRKCH (PKC eta)	Activity	Km app	20	5
EPHB4	Activity	Km app	19	5
FES (FPS)	Activity	Km app	19	1
IKBKB (IKK beta)	Activity	Km app	19	1
MAP2K1 (MEK1)	Activity	100	19	16
PRKCQ (PKC theta)	Activity	Km app	19	−4
ROS1	Activity	Km app	19	3
CSNK1G3 (CK1 gamma 3)	Activity	Km app	18	2
RAF1 (cRAF) Y340D Y341D	Activity	100	18	4
EGFR (ErbB1) T790M L858R	Activity	Km app	16	−1
PAK1	Activity	Km app	16	−1
PIM1	Activity	Km app	16	0
BRAF	Activity	100	15	11
AMPK (A1/B1/G3)	Binding		14	2
DYRK1B	Activity	Km app	14	3
KIT	Activity	Km app	14	−2
MAPK1 (ERK2)	Activity	Km app	14	6
DDR2	Binding		13	−1
MAPK3 (ERK1)	Activity	Km app	13	9
NTRK3 (TRKC)	Activity	Km app	13	1
AXL	Activity	Km app	12	0
DNA-PK	Activity	Km app	12	4
HCK	Activity	Km app	12	5
MAPK11 (p38 beta)	Activity	Km app	12	12
PDGFRA D842V	Activity	Km app	12	2
PLK2	Activity	Km app	12	11
CSK	Activity	Km app	11	10
PDK1	Activity	100	11	6
FGFR2	Activity	Km app	10	−4
FLT3 D835Y	Activity	Km app	10	5
HIPK3 (YAK1)	Activity	Km app	10	3
PDGFRA (PDGFR alpha)	Activity	Km app	10	0
AMPK (A2/B2/G3)	Activity	Km app	9	12
CDK1/cyclin B	Activity	Km app	8	5
DAPK3 (ZIPK)	Activity	Km app	8	0
DYRK2	Binding		8	1
FGFR3	Activity	Km app	8	1
IGF1R	Activity	Km app	8	2
IKBKE (IKK epsilon)	Activity	Km app	8	2
ERBB4 (HER4)	Activity	Km app	7	1
MAPK14 (p38 alpha) Direct	Activity	Km app	7	7
PRKACA (PKA)	Activity	Km app	7	16
DCAMKL1 (DCLK1)	Activity	Km app	6	7
EGFR (ErbB1) T790M	Activity	Km app	6	−2
EIF2AK2 (PKR)	Binding		6	12
FLT4 (VEGFR3)	Activity	Km app	6	4
FRAP1 (mTOR)	Activity	Km app	6	3
PAK4	Activity	Km app	6	2
RET	Activity	Km app	6	0
CSNK1G2 (CK1 gamma 2)	Activity	Km app	5	−2
KIT D816H	Binding		5	13
PRKCI (PKC iota)	Activity	Km app	5	−3
PRKCZ (PKC zeta)	Activity	Km app	5	6
PTK2 (FAK)	Activity	Km app	5	2
EGFR (ErbB1) L861Q	Activity	Km app	4	1
EPHA2	Activity	Km app	4	9
MAP2K2 (MEK2)	Activity	100	4	7
NIM1K	Activity	Km app	4	9
ROCK1	Activity	Km app	4	0
CDK2/cyclin A	Activity	Km app	3	11
CSNK1G1 (CK1 gamma 1)	Activity	Km app	3	6
DCAMKL2 (DCK2)	Activity	Km app	3	4
EGFR (ErbB1) L858R	Activity	Km app	3	−3
HIPK2	Activity	Km app	2	7
MAPK12 (p38 gamma)	Activity	Km app	2	4
MERTK (cMER)	Activity	Km app	2	4
MAPK13 (p38 delta)	Activity	Km app	1	14
TAOK2 (TAO1)	Activity	Km app	1	9
ERBB2 (HER2)	Activity	Km app	0	3
FLT1 (VEGFR1)	Activity	Km app	0	1
ZAP70	Activity	Km app	0	0
FGFR4	Activity	Km app	−1	8
TEK (Tie2)	Activity	Km app	−1	5
FLT3	Activity	Km app	−2	6
EGFR (ErbB1)	Activity	Km app	−4	2
AMPK (A1/B2/G1)	Binding		−5	2
AMPK (A2/B2/G1)	Binding		−5	5
ALK	Activity	Km app	−7	5
AMPK (A2/B2/G2)	Binding		−8	0
MAPK15 (ERK7)	Binding		−11	1
KIT V560G	Activity	Km app	−13	6
NTRK1 (TRKA)	Activity	Km app	−14	2
BMX	Activity	Km app	−22	7
PRKD1 (PKC mu)	Activity	Km app	−56	18

We thus focused our attention on MELK (*maternal embryonic leucine zipper kinase*). This kinase is described to be an oncogene and is overexpressed in multiple cancers, including melanoma^18,19^. Furthermore, MELK promotes mitosis and proliferation of melanoma cells via the NF-κB pathway^20^ and increases DNA damage tolerance to promote proliferation of breast cancer cells^21^.

As shown in Fig. 3B, a probe consisting in CRO15 labeled with a fluorescent tag showed a strong co-localization of CRO15 with MELK antibody (approximately 60%) in CT26 cells supporting an interaction between CRO15 with MELK. We next determined in vitro inhibition of MELK activation by CRO15 in comparison with a known MELK inhibitor, OTS167 (Fig. 3C). As OTS167, CRO15 decreases MELK activity in a dose-dependent manner. However, in contrast to CRO15, OTS167 is toxic to normal cells, such as melanocytes, keratinocytes, and fibroblasts (Supplemental Fig. 2C).

To determine whether MELK is a target of CRO15 in intact cells, we performed pull-down and biotin precipitation experiments in A375 cells infected with a MELK adenovirus and treated with a biotin-tagged CRO15. For the pull-down experiment, A375 lysates were incubated overnight with 100 μM biotin-CRO15 or with biotin-MTF465, a non-active CRO15 analog devoid of its pharmacophore. We thus demonstrated a direct interaction of MELK with biotin-CRO15 (Fig. 3D) but very weak with the negative control, biotin-MTF465. This result was further confirmed by detecting MELK after biotin precipitation on lysates of A375 treated for 2 h with 20 μM of biotin-CRO15.

We then determined the consequences of MELK inhibition for melanoma cells. Importantly, MELK expression correlates with melanoma progression^22^. A bio-informatic analysis of expression data on melanoma shows that MELK is significantly overexpressed in metastatic melanoma compared to primary melanoma (Supplemental Fig. 2B). Its overexpression correlates with the overexpression of its downstream target CDK1 (Cyclin-Dependent Kinase 1), implicated in the mitosis process. We performed kinetics of CRO15 (5 μM) in A375 cells, and detected a time-dependent decrease in expression of MELK but also of NF-κB phosphorylation and some mitosis markers, such as Cyclin E, CDK1, and CDK2 (Cyclin-Dependent Kinase 1 and 2) (Fig. 3E). Interestingly, we confirmed the inhibition of all actors implicated in the NF-κB pathway to induce melanoma cell proliferation (Supplemental Fig. 2A). Finally, MELK is known to increase DNA damage tolerance in breast cancer cells^21^. In our system, the inhibition of MELK by CRO15 was associated with a strong inhibition of the DNA damage repair pathway with decreased phosphorylation of ATM (Ataxia Telangiectasia Mutated) associated with an increase in the DNA damage marker γH2AX. CRO15-mediated activation of CHK2 (Checkpoint Kinase 2) and γH2AX were also observed by immunofluorescence in A375 cells (Supplemental Fig. 2D, E). To confirm that the biological activity of CRO15 involves the activation of AMPK and the inhibition of the MELK oncogene, we tried to counteract its effects on rescue experiments (Fig. 3G). Noticeably, knocking-down AMPK using siRNA or over-expressing MELK using adenoviral construct partially restored cell viability and the combination of both almost completely abolished the anti-melanoma effect of CRO15. This experiment unequivocally demonstrates the implication of both AMPK activation and MELK inhibition in the mode of action of CRO15.

### DNA damage repair pathway induces p53 pathway activation

**Fig. 4:**
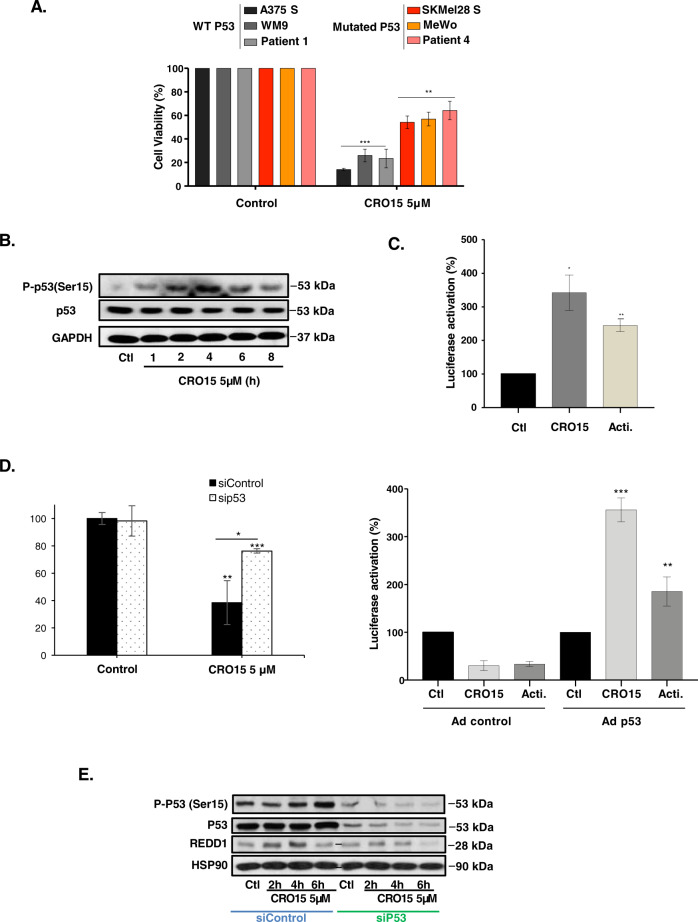
CRO15 treatment induces p53 pathway activation. **A** Indicated melanoma cell lines were treated with 5 μM CRO15 for 48 h before viable cells were counted using the trypan blue dye exclusion method. **B** A375 cells were treated with 5 μM CRO15 for the indicated duration, and lysates were analyzed by western blotting with indication antibodies. One representative experiment of three is shown. **C** Luciferase activity in A375 cells transfected with a p53 luciferase reporter upon 5 μM CRO15 treatment or actinomycin used as a positive control for p53 induction. **D** (left part) A375 cells were transfected with sip53 and then treated with 5 μM CRO15. After 24 h of treatment, viable cells were counted using the trypan blue exclusion method. The results are expressed as percentages of the DMSO control. (Right part) SK-MEL-28 cells were infected with control or p53-encoding adenoviruses and transfected with a p53 luciferase reporter. Cells were treated 24H with 5 μM CRO15 and a luciferase assay was performed. **E** A375 cells were transfected with control or p53 siRNA and treated with 5 μM CRO15 for the indicated duration. Lysates were analyzed by western blotting with the indicated antibodies. One representative experiment of three is shown. For **C** and **D**, data given as the means ± SEM of three independent experiments performed in triplicate. *p < 0.05; **p < 0.01; ***p < 0.001.

As described in the literature, the ATM/CHK2 axis is often responsible for p53 activation on serine 15 after DNA damage^23^. Furthermore, AMPK activators included metformin-activated p53 by phosphorylation on serine 15. Thus, we wanted to determine whether CRO15 can also activate this protein. We first examined the viability of different melanoma cells mutated for p53 after CRO15 induction and we observed that, in the cells expressing an inactive form of p53, the decrease of viability is less important than in the other melanoma cell lines expressing Wild Type p53 (Fig. 4A). We next wanted to determine whether p53 is implicated in CRO15 mechanisms. CRO15 induced a time-dependent phosphorylation of p53 on Ser15 that suggested p53 activation in A375 cells (Fig. 4B). We confirmed an increase in p53 activity upon CRO15 treatment by luciferase assays (Fig. 4C).

As expected, the activity of endogenous p53 was not detectable in response to CRO15 or positive control actinomycin in p53-mutated SK-MEL-28 cells (Fig. 4D, right part). When we infected these cells with wild type p53, CRO15 induced a strong activity of p53. In parallel, shutting down p53 expression with a siRNA induced a partial restoration of cell viability mediated by CRO15 (Fig. 4D, left part). This result is confirmed by western blot analysis, which showed that knockdown of endogenous p53 abolished the phosphorylation of p53 on Ser15 and attenuated the increase in expression of a direct target gene of p53, REDD1 (Regulated in Development and DNA damage response 1) in response to CRO15 (Fig. 4E).

Finally, SK-MEL-28 cells were infected with an adenovirus encoded an active form of p53, and we observed that when we restored p53 function, we restored the sensitivity to CRO15 (Supplemental Fig. 3A, B).

### CRO15 mediates the activation of autophagy and apoptosis in melanoma cells

**Fig. 5:**
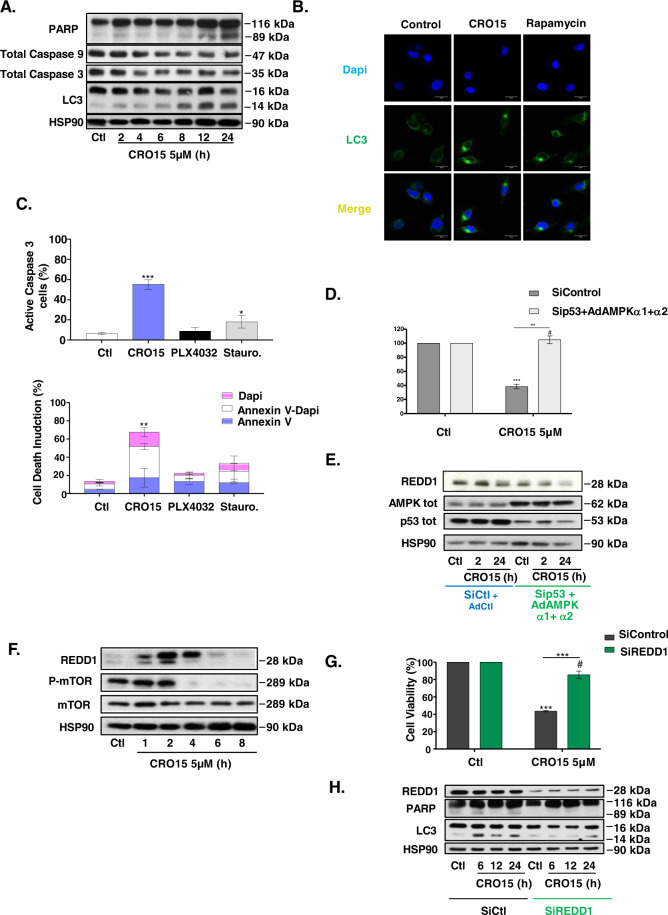
CRO15 mediates the activation of autophagy and apoptosis in melanoma cells. **A** A375 cells were treated with 5 μM CRO15 for the indicated duration. Lysates were analyzed by western blotting with the indicated antibodies. One representative experiment of three is shown. **B** Immunofluorescence pictures of A375 melanoma cells treated with DMSO, 5 μM CRO15 or 400 nM of the autophagy inducer rapamycin for 6 h. LC3B was labeled with antibody (green), and DNA was visualized with DAPI (blue). **C** A375 melanoma cells were treated for 24 h with 5 μM CRO15, 5 μM PLX4032 or with 1 μM of the pan-kinase inhibitor staurosporine for 6 h used as positive control of apoptosis induction. Cells were analyzed by flow cytometry using a specific antibody against Caspase 3 active protein conjugated with FITC (upper panel) or Annexin V conjugated with ALEXA and DAPI (lower panel) or against. *p < 0.05; **p < 0.01; ***p < 0.001. **D** A375 cells were infected with dominant negative adenoviruses against α1 and α2 AMPK subunits for 24 h before transfection with sip53 for another 24 h. Cells was determined by trypan blue staining after a 24 h treatment with 5 μM CRO15 or DMSO control. **E** A375 cells were infected with dominant negative adenoviruses against α1 and α2 AMPK subunits for 24 h and were transfected with sip53 for another 24 h. Cells were treated with 5 μM CRO15 for the indicated durations, and lysates were analyzed by western blot with the indicated antibodies. One experiment of three is shown. **F** A375 cells were treated with 5 μM CRO15 for the indicated durations, and lysates were analyzed by western blotting with the indicated antibodies. One representative experiment of three is shown. **G** A375 cells were transfected with siREDD1 for 24 h and then treated with 5 μM CRO15 for 24 h before cell viability was determined using the trypan blue exclusion method. **H**. A375 cells were transfected with siREDD1 for 24 h and then treated with 5 μM CRO15 for the indicated duration. Lysates were analyzed by western blotting with the indicated antibodies. One representative experiment of three is shown. For **D** and **G**, the results are expressed as percentages of the control and data given as the means ± SEM of three independent experiments performed in triplicate. *p < 0.05; **p < 0.01; ***p < 0.001.

Metformin induces both autophagy and apoptosis in melanoma cells to induce cell death^14^. We observed that CRO15 induced a time-dependent accumulation of LC3-II (Fig. 5A). This was confirmed by confocal microscopy, with accumulation of LC3 in melanoma cells under CRO15 stimulation in the same manner as that accumulation of LC3 under rapamycin stimulation, which is an autophagy activator (Fig. 5B and Supplemental Fig. 4A). To confirm that in our system, autophagy favors melanoma cell death and does not protect them, we blocked the autophagic flux with a protease inhibitor, pepstatin and a cysteine protease inhibitor, E64d. After stimulation with CRO15 for 24 h, we observed a partial restoration of melanoma cell viability when autophagy was blocked (Supplemental Fig. 4B, C). Finally, it was also shown by autophagy inhibition with siRNA against LC3 and ATG5 (Data not shown). For the apoptosis pathway, we observed a characteristic cleavage of PARP (Poly-ADP-Ribose polymerase) in a time-dependent manner under CRO15 treatment (Fig. 5A). We also observed a decrease in total caspase 3 and 9 expression, which are specific markers for caspase-dependent apoptosis. To confirm the implication of apoptosis in CRO15-induced cell death, we performed flow cytometry analysis (Fig. 5C). First, we examined the presence of annexin V on the cellular membrane in melanoma cells. Indeed, when cells enter apoptosis, a flip-flop into the plasma membrane takes place and phosphatidylserines, which are normally inside the cells, are exposed to the extracellular medium and can link with annexin V. After CRO15 stimulation, we observed a large increase in melanoma cells stained with annexin V. We also studied the presence of caspase 3 active cells after CRO15 stimulation. We observed that melanoma cells treated with this drug present approximately 60% of caspase-3 active cells compared to the control. Finally, autophagy and apoptosis after CRO15 stimulation were also observed in electronic microscopy (Supplemental Fig. 4D). In these pictures, we observed the formation of apoptotic blebs and autophagolysosomes with a double membrane characteristic of autophagy.

To determine whether these two mechanisms are dependent on each other, we blocked autophagy by siLC3, apoptosis by Q-VD (inhibitor of pan caspase) and both mechanisms at the same time (Supplemental Fig. 4E, F). We observed that when the two mechanisms are blocked, all melanoma cell viability is restored. When we blocked only autophagy or apoptosis, we partially restored melanoma cell viability. Interestingly, when the two mechanisms are blocked independently, the other mechanism is still activated. Thus, autophagy and apoptosis are two mechanisms activated independently by CRO15.

We identify pathways involving AMPK and/or p53, which are responsible for the cell death mechanism induced by CRO15. To confirm that only these two proteins are responsible for melanoma cell death induced by CRO15, we blocked these two proteins and studied cell viability. After infection with adenoviruses of dominant negative forms of AMPKα1 and α2 and transfection of sip53, we observed a total restoration of melanoma cell viability after CRO15 stimulation for 24 h (Fig. 5D, E), indicating that these 2 proteins are responsible for cell death induced by CRO15. Interestingly, by western blot analysis, we observed that CRO15 increased the expression of REDD1 after 2 h of stimulation but that this increase was lost when the AMPK and p53 pathways were inhibited (Fig. 5E). Thus, we wanted to determine whether REDD1 can be implicated in melanoma cell death mechanisms induced by CRO15. REDD1 is a protein implicated in the stress response (i.e., DNA damage, metabolic dysregulation, etc.) and can activate different cell death mechanisms when restoration of melanoma cell viability stress is too strong for the cell. We observed that REDD1 is activated by CRO15 at an early time point post-stimulation and that its principal target, mTOR, is no longer phosphorylated and therefore inactivated (Fig. 5F). To confirm the implication of REDD1 in the cell death mechanism, we inhibited REDD1 expression with siRNA, and in blue trypan exclusion, we observed that there was a total restoration of melanoma cell viability after 24 h of CRO15 stimulation (Fig. 5G). Interestingly, we showed in western blot analysis that when REDD1 is inhibited, autophagy (with LC3 II conversion) and apoptosis (with PARP cleavage) are lost under CRO15 stimulation (Fig. 5H).

In conclusion, we demonstrate that CRO15 induces melanoma cell death by AMPK and p53 pathways to induce autophagy and apoptosis of melanoma cells, and this process is REDD1 dependent.

### CRO15 decreases tumor growth in mouse models

**Fig. 6:**
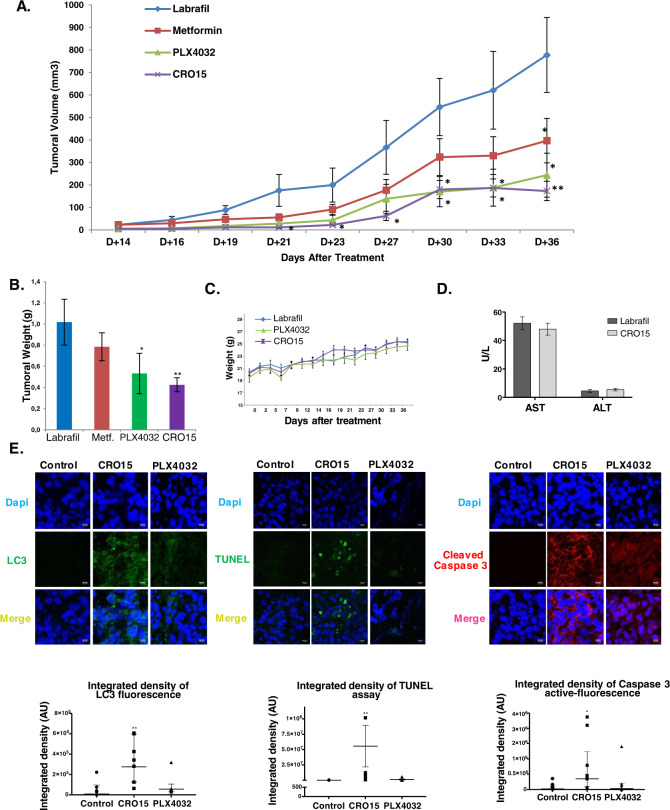
CRO15 decreases tumor growth in mouse models. **A** Female immune-deficient BALB/c nu/nu (nude) mice were inoculated subcutaneously with 1.0 × 10^6^ A375 cells. After 4 days, mice (*n* = 6 in each group) were treated with CRO15 (0.7 mg/mouse/day), PLX4032 (0.7 mg/mouse/day) or vehicle (Labrafil). Tumor growth curves were determined by measuring the tumor volume. The bars indicate the mean ± SEM. *p < 0.05; **p < 0.01. **B** Tumor weight of A375 xenograft after mouse euthanasia. The bars indicate the mean ± SEM *p < 0.05. C. Mice weight over the course of the experiment. **D** Quantification of hepatic transaminases (AST and ALT) on plasma of nude mice treated with vehicle or CRO15 at end point. **E** Immunofluorescence staining on xenograft tumor sections. LC3B (green) and active caspase 3 (red) were immunolabeled, and apoptotic cells were visualized with a TUNEL kit. Nuclei were visualized with DAPI (blue). Integrated density was determined for each condition on Fuji. Data are geometric means with 95% CI of three independent experiments performed in triplicate. *p < 0.05; **p < 0.01; ***p < 0.001.

To assess the antineoplastic potential effect of CRO15 in vivo, A375 melanoma cells (1.0 × 10^6^) were injected subcutaneously into 5-week-old female athymic nude mice. After tumor apparition (approximately size of 50 mm^3^), mice were treated every day with Labrafil (control), PLX4032 (0.7 mg/mouse/day) and CRO15 (0.7 mg/mouse/day) over a period of 4 weeks. This dose corresponds to the equivalent dose of PLX4032 given to patient in melanoma therapy. Untreated control mice rapidly developed visible tumors, and we observed a dramatic increase in tumor growth throughout the study (Fig. 6A). By contrast, treatment of mice with PLX4032 or CRO15 markedly attenuated the development of tumors. Moreover, the tumors derived from CRO15-treated mice present consistently and significantly lower weight than those from untreated control mice (Fig. 6B). Furthermore, the treatment of mice with CRO15 induced no apparent toxicity, and we noted no change in their behavior and body mass (Fig. 6C). Furthermore, we evaluated hepatic toxicity by measuring hepatic transaminases in the sera of treated mice (Fig. 6D), and no change appeared between control and CRO15-treated mice, indicating the absence of hepatic toxicity. We then investigated the molecular mechanisms involved in the anti-tumorigenic effects of CRO15 in vivo. Tumor sections from mice were stained with LC3 antibody, and we observed an increase in LC3 expression in CRO15 conditions, confirming the activation of autophagy by CRO15 in vivo (Fig. 6E). For apoptosis, we stained the tumor sections with active-caspase 3 antibodies and TUNEL assay. We observed an increase in both active caspase 3 and TUNEL staining in CRO15 conditions (Fig. 6E). These results confirmed the activation of autophagy and apoptosis in vivo by CRO15.

### CRO15 induces cell death in melanoma cells resistant to BRAF inhibitors

**Fig. 7:**
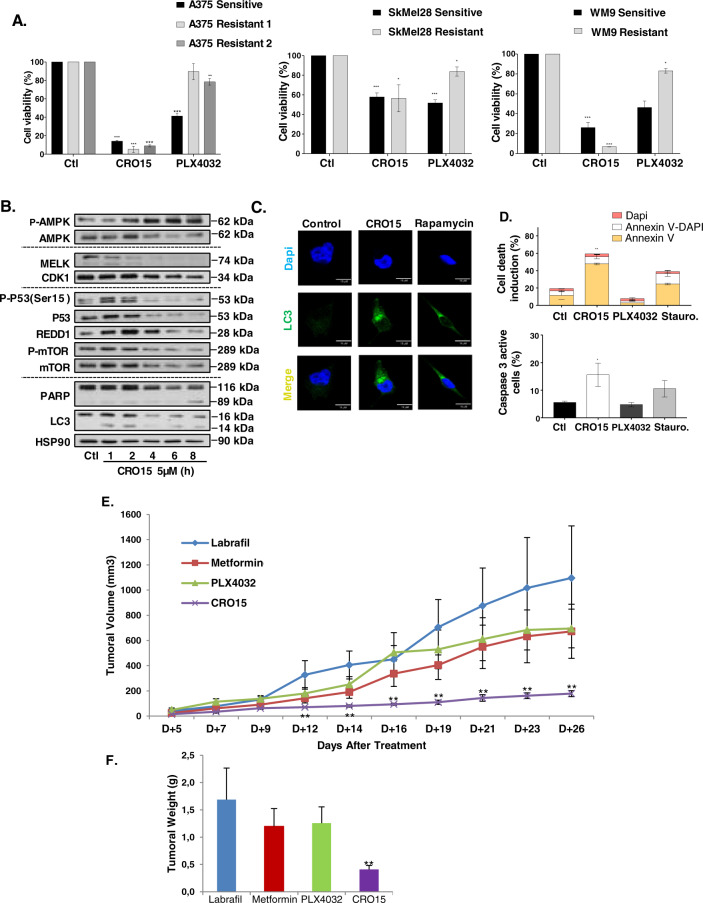
CRO15 induced death in melanoma cells resistant to BRAF inhibitors. **A** Indicated melanoma cell lines were treated with 5 μM CRO15 or 5 μM PLX4032. After 48 h, cell viability was determined by Trypan blue exclusion method. The results are expressed as percentages of the corresponding control and data given as the means ± SEM of three independent experiments performed in triplicate. *p < 0.05; **p < 0.01; ***p < 0.001. **B** A375-resistant cells were treated with 5 μM CRO15 for the indicated durations. Lysates were analyzed by western blotting with the indicated antibodies. One representative experiment of three is shown. **C** Immunofluorescence images of A375-resistant melanoma cells treated with 5 μM CRO15 or 400 nM rapamycin for 6 h or DMSO control. LC3B was labeled with antibody (green), and the nuclei were visualized with DAPI (blue). **D** A375-resistant melanoma cells were treated for 24 h with 5 μM of CRO15 or PLX4032 for 24 h or with 1 μM staurosporine for 6 h. Cells were analyzed in flow cytometry with a specific antibody directed Annexin V (conjugated with ALEXA) or Caspase 3 active protein (conjugated with FITC). *p < 0.05; **p < 0.01; ***p < 0.001. **E** Female immune-deficient BALB/c nu/nu (nude) mice were inoculated subcutaneously with 1.0 × 10^6^ A375-resistant cells. After 7 days, mice (*n* = 6 in each group) were treated with CRO15 (0.7 mg/mouse/day), PLX4032 (0.7 mg/mouse/day) or vehicle (Labrafil). Tumor growth curves were determined by measuring the tumor volume. The bars indicate the mean ± SEM. *p < 0.05; **p < 0.01. **F** Tumor weight of A375-resistant xenograft after mouse euthanasia. The bars indicate the mean ± SEM *p < 0.05; **p < 0.01.

As mentioned above, treatment of BRAF V600E melanoma patients with BRAF inhibitors, such as PLX4032, leads to systematic resistance and metastasis relapse. In Fig. 7A, we showed that CRO15 induces melanoma cell death in A375, SK-MEL-28 and WM9 melanoma cells that had acquired resistance to PLX4032. Interestingly, we confirmed that in SK-MEL-28 cells mutated for p53, CRO15 was less efficient. In addition, we observed that CRO15 induces the same molecular and death mechanisms in A375 resistant melanoma cells. Indeed, in western blot analysis, we observed phosphorylation of AMPK after CRO15 stimulation. We also observed a decrease in MELK expression and CDK1 in a time-dependent manner. Phosphorylation of p53 (ser15), increased expression of REDD1 and decreased phosphorylation of mTOR were confirmed at early time of stimulation by CRO15 at 5 μM (Fig. 7B). For cell death mechanisms induced by CRO15, PARP cleavage and LC3-II accumulation were observed in western blot analysis (Fig. 7B). These two mechanisms were confirmed by confocal microscopy with staining of LC3 for autophagy (Fig. 7C) and flow cytometry, with increasing annexin V and active caspase 3 staining for apoptosis (Fig. 7D). Finally, we tested the anti-tumorigenic effect of CRO15 in mice injected with A375 R melanoma cells presenting acquired resistance to the BRAF inhibitor PLX4032. Briefly, as previously described, we subcutaneously injected A375 Resistant cells (1.0 × 106) into nude mice, which were then treated with Labrafil, PLX4032 (0.7 mg/mouse/day) and CRO15 (0.7 mg/mouse/day). Untreated mice or PLX4032-treated mice rapidly developed dramatic tumor growth throughout the study (Fig. 7E). In contrast, CRO15-treated mice markedly attenuated the development of tumors. Moreover, tumors derived from CRO15-treated mice were consistently and significantly lower than those from untreated or PLX-treated mice (Fig. 7F). These data clearly demonstrate the anti-melanoma activity of CRO15 in vivo in BRAF inhibitor-resistant cells. As observed in tumors from melanoma cells sensitive to BRAF inhibitor, CRO15 induced a strong increase in LC3, active caspase 3 and TUNEL staining compared to tumor sections from control or PLX4032 (Supplemental Fig. 5B). In this experiment, no apparent toxicity appeared with no change in mouse behavior, body mass (Supplemental Fig. 5A) or liver mass (data not shown).

## Discussion

Currently, therapies against metastatic melanoma are not efficient enough. Indeed, targeted therapies against BRAF V600E/MEK initially show very efficient responses, but after a few weeks of treatment, melanoma relapses and metastases begin to grow again^3–6,11^. For immunotherapies, objective responses are sustainable but only in 30 to 40% of patients in the best way^7,8,24^. Thus, for many patients (more than 50%), there are no effective treatments for their melanoma, and discovering new therapies is essential. In this report, we present anti-melanoma effects of a new biguanide-derived compound CRO15. Melanoma presents many different mutations and oncogene proteins. In this study, we showed that CRO15 induces melanoma cell death independently of their mutational status, and this is due to activation of the AMPK and p53 pathways and inhibition of MELK kinase activity. As biguanides, CRO15 is able to activate the AMPK pathway by disrupting mitochondrial metabolism and by changing the ATP/AMP ratio. Interestingly, in melanoma glycolytic metabolism, mediated by mitochondrial activity, is very important^25,26^. Furthermore, it was described that melanoma cells resistant to BRAF inhibitors showed an oxidative metabolism that was more important and dependent on mitochondria for cell survival^27^. In this context, mitochondria seem to be very important, and their pharmacological dysregulation could pave the road to develop new melanoma therapies. Furthermore, AMPK is an energy sensor of the cells; it plays a role in cellular energy homeostasis. It is composed of 3 subunits α, β, and γ^28^. Both subunits play a specific role in the stability and activity of AMPK, and the α subunits play a role in catalytic activity^29^. AMPK plays an important role in metabolism regulation with inhibition of fatty acid synthesis, cholesterol and triglycerides by ACC phosphorylation^30^. However, AMPK also plays a role in the induction of autophagy and apoptosis by a number of different mechanisms. For example, AMPK can directly phosphorylate ULK1 (UNC-51-like kinase)^31^ or activate REDD1^32^, both of which dephosphorylate mTOR and induce autophagy. Interestingly, AMPK can also activate the apoptosis pathway by eIF2a (eukaryotic Initiation Factor 2 α)^33^ or by p53^34^. In our study, we showed that the AMPK pathway is partially responsible for melanoma cell death induced by CRO15. Despite the fact that the AMPK pathway is present in all human cells and in cancer human cells, but we did not observe any toxicity in normal human cells. Interestingly, we observed that CRO15 is able to induce minimal AMPK phosphorylation in both normal keratinocytes and melanocytes without affecting their viability. Our hypothesis is that the AMPK pathway is constitutively more activated in melanoma cells because of their metabolic activity. When CRO15 is added to these cells, a threshold is exceeded, and this high level of AMPK induces the activation of autophagy and apoptosis in melanoma cells. In normal human cells, the level of AMPK is lower than this threshold, and thus, no cellular death is induced. However, the other scenario is that cancer cells might overexpress CRO15 transporters.

To determine the AMPK-independent effects of CRO15, we performed a kinome assay, and we identified 2 kinases significantly inhibited by our compound. The first candidate is a member of the Ser/Thr protein kinase family, MAPKAPK2. This kinase is regulated through direct phosphorylation by p38 MAP kinase. In conjunction with p38 MAP kinase, this kinase is known to be involved in many cellular processes, including stress and inflammatory responses, nuclear export, gene expression regulation, cell proliferation and metastasis invasion. Heat shock protein HSP27 was shown to be one of its substrates^35,36^. Our experiments quickly allowed us to eliminate this candidate in the CRO15 mode of action in melanoma cells. Indeed, CRO15 rapidly increases the activation of p38 and does not decrease the phosphorylation of MAPKAPK2 and of its substrate HSP27. Furthermore, our preliminary experiments indicate that CRO15 is not able to inhibit melanoma cell invasion, which constitutes one of the main effects of the enzyme (data not shown)^36–38^.

The second evaluated candidate of CRO15 was the oncogene MELK. This protein is a member of the AMPK/Snf1 family, which is a serine/threonine kinase that is highly conserved across a variety of mammalian and non-mammalian species. MELK has been shown to be activated by auto-phosphorylation in vitro, which is a unique mechanism among the AMPK/Snf1 family members^39^. This kinase is overexpressed in several human cancers and stem cell populations, including melanoma cells^19,40,41^. MELK plays a prominent role in cell cycle control, cell proliferation and cancer treatment resistance and is a good candidate for potential anti-cancer therapies. Further, a recent study demonstrated the importance of MELK expression and its effects on the NF-κB pathway in melanoma proliferation^20^. Indeed, MELK is overexpressed in primary and even more in metastatic melanoma, suggesting its implications in melanoma progression^22^. This increasing expression is correlated with increased expression of CDK1, an essential protein promoting proliferation and mitosis^42^. However, two recent articles questioned the role of MELK in cancer development since, contrary to siRNA, disruption of MELK using the CRISP-Cas9 approach had no appreciable impacts on tumor cell growth^43,44^. This apparent discrepancy could be explained by a conditional dependency on MELK for the proliferation of cancer cells^45^ as it is required for clonogenic cell growth like other oncogenes such as MYC or KRAS.

Our results showed that CRO15 directly decreases MELK kinase activity during in vitro kinase assay but also in intact melanoma cells, as indicated by the inhibition of NF-κB phosphorylation and some mitotic makers, such as Cyclin E, CDK1 or CDK2. These results suggest that in our system, inhibition of MELK induced a decrease in mitosis and cell proliferation after CRO15 stimulation mediated by NF-κB pathway inhibition. Interestingly, in contrast to the other MELK inhibitor OTS167, CRO15 is non-toxic in normal cells, such as fibroblasts, melanocytes and keratinocytes. MELK is also implicated in the DNA damage response^21^. Indeed, MELK was shown to increase the tolerance of cancer cells to the DNA damage response by inhibiting the phosphorylation of ATM. This allows cancer cells to inhibit activation of death mechanisms induced by this response. When melanoma cells are treated with CRO15, ATM phosphorylation is observed during early stimulation, which induces activation of the DNA damage pathway response. These results suggest that CRO15 could remove ATM inhibition induced by MELK and reactivate the DNA damage response pathway. This activation allows phosphorylation of p53 on ser15 by CHK2, which leads to the activation of REDD1^46^, which is responsible for melanoma cell death. Indeed, p53 plays an essential role in human cells and is implicated in the DNA damage response induced by ATM^23^. Activation of p53 induced a protective response against DNA damage and tried to repair it. However, sustainable activation of DNA damages induced an increase in REDD1 expression by p53 and thus activation of cell-death mechanisms. REDD1 (RTP801/Dig2/DDIT4) was identified as a hypoxia-inducible factor 1 (HIF-1) target gene involved in the regulation of cell survival. REDD1 is also regulated in response to DNA damage, nutrient depletion, glucocorticoid and insulin^47,48^. This protein is a negative regulator of mTOR and is defined as a key metabolic regulator suppressing tumorigenesis thought effects on mTOR activity^49^ and mitochondria. After CRO15 treatment, REDD1 expression is quickly increased and induces mTOR dephosphorylation. This is responsible for autophagy induction, which is confirmed by the loss of LC3-II conversion when REDD1 is deleted. In addition, REDD1 also activates apoptosis^50^ and the knock down of REDD1 leads to loss of PARP cleavage in melanoma cells upon CRO15 treatment. REDD1 is the key protein activated in melanoma cells in response to CRO15 treatment and is responsible for melanoma cell death.

When the AMPK and p53 pathways are independently inhibited, activation of REDD1 by CRO15 is still conserved at a lower level. The fact that REDD1 can be independently activated by these two pathways is an advantage for CRO15 treatment. Indeed, many melanoma cells and other cancer cells present p53 inactivation. However, CRO15 can induce cell death even in p53-mutated melanoma cells. Moreover, MELK is important in melanoma progression and therefore may be implicated in resistance against current therapies. Indeed, we observed that MELK, as well as CDK1, is significantly overexpressed in metastatic melanoma. We next confirmed the efficiency of CRO15 in resistant melanoma cells. Counterintuitively, the decrease in viability induced by CRO15 seems to be more dramatic in PLX4032-resistant cells than in their parental counterpart, except for the SK-MEL-28 cell line, mutated for p53. The same mechanisms are activated in melanoma cells resistant to BRAF inhibitors, which implicate MELK, AMPK, p53, and REDD1. Autophagy and apoptosis induced by CRO15 are responsible for melanoma cell death after REDD1 activation in these resistant melanoma cells. Furthermore, MELK is overexpressed in many cancers, such as breast cancer, glioma, and colon cancer, and plays a role in oncogenes in these cancers^18,19,41^. Interestingly, CRO15 can decrease the viability of a number of cancer cell lines, including breast, cervix or prostate cancer (Supplemental Fig. 6).

Finally, in vivo results confirmed our hypothesis that melanoma cells are impacted by CRO15 treatment. Indeed, effects on tumoral volume and tumoral growth both in xenograft-sensitive and resistant to BRAF inhibitor mice models show that CRO15 is able to induce melanoma cell death without apparent toxicity in mice. Activation of autophagy and apoptosis in these tumors confirms the implications of the same pathways in vivo. Metabolic activity of melanoma cells and the potential overexpression of MELK in these cells seem to be important for CRO15-induced cell death and could explain why normal human or mouse cells are not affected by this compound. All these results allow us to think that CRO15 could be a good candidate in the development of new treatments against melanoma or other cancer types. Furthermore, a recent study by Janostiak et al. showed that in melanoma cells, MELK expression is in part increased by the MAPK kinase pathway via transcription factor E2F1 (19). Thus, inhibition of MELK in combination with BRAF inhibitors allows for an effect on resistant melanoma cell viability compared to BRAF inhibitors alone and decreases the development of resistance. In this context, it could be interesting to study the effect of the combination of CRO15 or other MELK inhibitors and BRAF inhibitors for melanoma treatment.

In summary, we demonstrate that CRO15 inhibits tumor growth through autophagic and apoptotic mechanisms induced by REDD1 and initiated by AMPK and p53 activation by inhibition of MELK kinase activity. Considering the drastic effects of CRO15 on cancer growth in mice, together with the lack of apparent toxicity, this study provides compelling data to support the future evaluation of CRO15 in clinical trials and strengthens the idea that MELK inhibition could be useful, alone or in combination, as a new therapeutic approach in cancer treatment.

## Materials and methods

### Reagents and antibodies

Trypan blue, DMEM, RPMI, penicillin/streptomycin, trypsin, siRNA against LC3, MELK and p53 were purchased from Life Technology. Fetal calf serum (FCS) was purchased from HyClone. MCDB 153 medium, metformin, staurosporine and dimethylacetamide were purchased from Sigma-Aldrich (Saint Quentin Fallavier, France). Labrafil M1944 Cs was purchased from Gatte fosse. Annexin V and DAPI were purchased from Roche Applied Science. PLX4032 was purchased from Euromedex. HSP60, p53, HSP90, Actin, Cyclin E, AMPKα1 and AMPKα2 antibodies were purchased from Santa Cruz Biotechnology (TEBU; Le Perray en Yvelines, France). LC3-B, Ⓟ-AMPKα (T172), Ⓟ-mTOR (S2448), PARP, AMPKα total, mTOR total, Ⓟ-ACC (Ser79), ACC total, MELK, CDK1, Ⓟ-CHK2 (Thr68), CHK2 total, CDK2, Ⓟ-P53 (ser15), Caspase 9 and Caspase 3 antibodies were from Cell Signaling Technology (Berverly, MA, USA). Antibodies against Ⓟ-CDC25B (S323), CDC25B total, gamma-H_2_AX, and ATM total were purchased from ABCAM. Antibodies against cyclin D1 and pRb were from BD Bioscience (Pont de Claix, France). REDD1 antibody was purchased from Proteintech. TMRE was purchased from Abcam (ab113852-TMRE Mitochondrial Membrane Potential Assay Kit). ATP production was measured using a Malachite Green Phosphate Assay Kit produced by Cayman chemical (n°10009325). MELK adenovirus and Ⓟ-ATM (10H11.E12) antibody were purchased from TEBU.

### Cell cultures

Normal human melanocytes and fibroblasts were prepared and maintained as previously described^51^. Melanoma cell lines were purchased from the American Tissue Culture Collection. The resistant melanoma cell lines A375 and SK-MEL-28 were a gift from Professor P. Marchetti and described in Corazao-Rozas et al.^52^. See Supplemental Experimental Procedures for cell preparation.

### siRNA transfection

Transfection of duplex siRNAs (50 nmol/l) was carried out using Lipofectamine RNAiMAX (Invitrogen) in OptiMEM (Invitrogen) as described^53^. The day after transfection, CRO15 was added to the medium, and proteins were extracted 24 h. siRNA targeting LC3, P53 and MELK were purchased from Life Technology. REDD1 siRNA was purchased from Dharmacon (Lafayette, CO, USA). As a nonspecific control, scramble sequence of siRNAs were used.

### Adenoviral infections

Adenoviruses encoding a dominant-negative form (Ad AMPK-DN) of subunits α1 and α2 of AMPK were a generous gift of Dr. Foufelle (INSERM, UMR-S 872, Paris, France). Adenoviruses encoding MELK and p53 protein have been purchased from Tebu. An adenovirus of which the expression cassette contains the major late promoter with no exogenous gene was used as a control (Ad control). Adenoviruses were produced in human embryonic kidney 293 cells and stored at −80 °C. A375 cells were infected for 48 h with adenoviruses before CRO15 treatment.

### Trypan blue exclusion assay

For trypan blue staining, 200 μl of cells were aseptically transferred to a 1.5 ml clear Eppendorf tube and incubated for 3 min at room temperature with an equal volume of 0.4% trypan blue solution. Viable cells were counted, and the results are expressed as the percentage of control cells. All the experiments were performed 3 times in triplicate.

### Western blot analysis

Western blot analyses were performed as described^54^. Proteins were extracted in buffer containing 50 mmol/l Tris-HCl (pH 7.5), 15 mmol/l, NaCl, 1% Triton X-100, and 1× protease and phosphatase inhibitors. Briefly, after protein dosage by Pierce BCA protein assay kit, 30 μg of cell lysate from each condition were separated by SDS-PAGE, transferred onto a polyvinylidene fluoride membrane (Millipore), and then exposed to the appropriate antibodies. Proteins were visualized with the ECL System from Amersham. The western blot analyses shown are representative of at least three independent experiments.

### Immunofluorescence microscopy

See Supplemental Experimental Procedures

### SeaHorse experiments

ECAR and OCR were measured using the Seahorse XF96 Extracellular Flux Analyzer (Seahorse Bioscience, North Billerica, MA, USA). The Cell Mito Stress Test media supplemented with 2 mm Glutamax, 1 mm sodium pyruvate, and 25 mm glucose was used. Briefly, 2. 10^4^ cells were plated in a seahorse-specific 96-well plate and then pre-treated for 6 h with 5 μM CRO15 or 10 mM metformin or control.

### Mitochondrial transmembrane potential measuring

See Supplemental Experimental Procedures

### Cell fractionation

Cell fractionation was performed with the Calbiochem kit, proteoextract 539790 following manufacturer’s instructions.

### Kinome assays

Kinome assays were performed with a kit as described in the Thermo Fisher Kit, SelectScreen^TM^ Biochemical Profiling Service, Z′-LYTE^TM^ Assay.

### MELK kinase assay

MELK kinase assay was performed with the ADP-Glo™ Kinase Assay kit from Promega.

### Pull-down assay

Cells or cell lysates were incubated with CRO15-biotin or control overnight at 4 °C. Fifty microliters of streptavidin-agarose were added for 2 h at 4 °C. The CRO15/MELK complexes were analyzed by SDS-PAGE and immunoblotted using anti-MELK antibody.

### Bioinformatic analysis

See Supplemental Experimental Procedures

### Luciferase assay

See Supplemental Experimental Procedures

### Flow cytometry

See Supplemental Experimental Procedures

### In vivo murine cancer model

See Supplemental Experimental Procedures

### Circulating levels of transaminase

Determination of serum transaminases (aspartate aminotransferase/alanine aminotransferase) was performed using in vitro tests with pyridoxal phosphate activation on a Roche/Hitachi cobas c systems (ASTPM, ALTPM, cobas) as previously described^12^.

### Electron microscopy

A375 cell pellets were collected in 1% OsO4, dehydrated in alcohol series, and embedded in epoxy resin. Thin sections were contrasted with uranyl acetate and lead citrate. Preparations were observed either with a Philips CM12 electron microscope operating at 80 kV (FEI) or with a Jeol 1400 mounted with CCD cameras (Morada, Olympus SIS).

### ATP production measurement

Cells were plated on a 6-well plate and treated with 5 μM CRO15 for 6 h or with control. At the end of stimulation, cells were lysed in FISHER buffer (TRIS 2 M pH = 8, NaCl 5 M, NP-40 1%, Proteases Inhibitors), and ATP production was measured as described in the malachite green phosphate assay protocol by absorbance at 620 nm.

### Autophagic flux

Cells were pre-treated for 2 h with pepstatin and E64d to inhibit autophagic flux prior to treatment for 24 h with 5 μM CRO15, 400 nM rapamycin or control. Cells were analyzed by blue trypan exclusion and western blot analysis.

Unless specified in figure legends, all the data were subjected to two-tailed T-test analysis using Prism 7 (GraphPad Software, San Diego, California, USA). The results are presented as the means ± SEM.

## Supplementary information

Supplemental informations

Supplemental Figure 1

Supplemental Figure 2

Supplemental Figure 3

Supplemental Figure 4

Supplemental Figure 5

Supplemental Figure 6
