# Supplementary material for: Discovery of a new molecule inducing melanoma cell death: dual AMPK/MELK targeting for novel melanoma therapies
Source: Cell Death Dis. 2021 Jan 11;12(1):64. doi: 10.1038/s41419-020-03344-6 (PMC7801734; doi:10.1038/s41419-020-03344-6)
Supplement: Supplementary file 1 — Supplemental informations [file 41419_2020_3344_MOESM1_ESM.docx]

**SUPPLEMENTAL INFORMATIONS**

**Discovery of a new molecule inducing melanoma cell death: dual AMPK/MELK targeting for novel melanoma therapies**

Emilie Jaune^1,2*^, Elisa Cavazza^1,2*^, Cyril Ronco2^3,2^, Oleksandr Grytsai^3,2^, Patricia Abbe^1,2^, Nedra Tekaya^1,2^, Marwa Zerhouni^1,2^, , Guillaume Beranger^1,2^, Lisa Kaminski^4,2^, Frédéric Bost^4,2^, Maeva Gesson^1,2^, Meri Tulic^1,2^, Paul Hofman^5,6,7^, Robert Ballotti^8,2^, Thierry Passeron^1,9^, Thomas Botton^1,2^, Rachid Benhida^3,2^, Stéphane Rocchi^1,2^.

^1^ INSERM, U1065, Equipe 12, Centre Méditerranéen de Médecine Moléculaire (C3M), Bâtiment ARCHIMED, 151 route de saint Antoine de Ginestière, 06204 Nice cedex 3, France.

^2^ Université Cote d'Azur, Nice, France

^3^Institut de Chimie de Nice UMR-CNRS 7272, Université Nice Sophia Antipolis, Parc Valrose, 06108 Nice Cedex 2, France

^4^ INSERM, U1065, Equipe 5, Centre Méditerranéen de Médecine Moléculaire (C3M), Bâtiment ARCHIMED, 151 route de saint Antoine de Ginestière, 06204 Nice cedex 3, France.

^5^UFR de Médecine, Université de Nice Sophia Antipolis, 06000 Nice, France

^6^Institute of Research on Cancer and Ageing of Nice (IRCAN), INSERM U1081, CNRS UMR7284, Nice 06107, France

^7^Laboratoire de pathologie clinique et expérimentale et Hospital-related biobank (BB-0033-00025), Hôpital Pasteur, 06002 Nice, France

^8^ INSERM, U1065, Equipe 1, Centre méditerranéen de Médecine Moléculaire (C3M), Bâtiment ARCHIMED, 151 route de saint Antoine de Ginestière, 06204 Nice cedex 3, France.

^9^ Service de Dermatologie, Hôpital Archet II, CHU, 06204 Nice, France.

Running title: New anti-melanoma compounds

Conflict of Interest statement: There is no conflict of interest

*Equal contribution to this work

Correspondence should be address to:

[srocchi@unice.fr](mailto:srocchi@unice.fr) (S.R.)

[benhida@unice.fr](mailto:benhida@unice.fr) (R.B.)

[Thomas.BOTTON@univ-cotedazur.fr](mailto:Thomas.BOTTON@univ-cotedazur.fr) (T.B.)

**EXPERIMENTAL SUPPLEMENTAL PROCEDURES**

*Cell Cultures*

Cells were grown in RPMI 1640 or in DMEM supplemented with 10% FCS and penicillin/streptomycin (100 U/ml/50 mg/ml) at 37°C and 5% CO_2_. Fresh sterile tissues were obtained from surgical waste from patients diagnosed with metastatic melanoma at the Nice CHU hospital and treated as previously reported (Lehraiki et al., 2015). Written informed consent was obtained from each patient included in this study, and the study was approved by the hospital’s ethics committee (Nice Hospital Center and University of Nice Sophia Antipolis, no. 210-2998).

*Immunofluorescence Microscopy*

Monolayers prepared for fluorescent staining were grown on glass coverslips. Immunofluorescence experiments were carried out as previously described (Rouaud et al., 2018). The confocal microscope was a Nikon A1R on an inverted Nikon Eclipse Ti stand (Nikon Instruments, Japan) using objectives Plan 2X dry, 0.1 NA and/or Plan Fluor 10X dry NA 0.30 and/or Plan Apo 20X NA 0.8 and/or Apo 40X water 1.3 NA and/or Plan Fluor 40X oil 1.3 NA and/or Plan Apo 63X oil 1.4 NA. The LASERs used were LASER diode 405 nm and/or Argon LASER 488 nm and/or DPSS 561 nm, HeNe 633 nm. The microscope was composed of 2 PMTs equipped with 450/50 and 700/75 filter set, 2 GaAsP equipped with 515/30 and 585/65 filter-set, and 1 external PMT for transmission. The microscope was equipped with an automated xy stage for mosaic acquisitions (Märzhäuser, Wetzlar, Germany), and z-acquisitions were performed using the microscope z-drive and/or piezo stage.

For each condition, a minimum of 30 cells or 3 sections of tumors were analysed. Quantification of integrated intensity by cells was analyzed as a macro on Fiji.

*Mitochondrial Transmembrane Potential Measurement*

Mitochondrial transmembrane potential was measured by TMRE (tetramethylrhodamine, ethyl ester) staining. TMRE is a cell-permeant, positively charged red-orange dye that readily accumulates in active mitochondria due to their relative negative charge. Depolarized or inactive mitochondria have decreased membrane potential and fail to sequester TMRE. Cells were plated in 6-well plates and treated with 5 μM CRO15 for 6H, with 10 mM metformin for 6, with DMSO as a negative control or with 20 μM CCCP for 20 mins as a positive control. Then, 50 nM TMRE was added for 20 min at 37°C. Cells were washed, resuspended in PBS/BSA buffer and analyzed at Ex/Em = 549/575 nm by flow cytometry.

*Bioinformatic Analysis*

Melanoma gene expression microarray datasets are downloaded from Gene Expression Omnibus (GEO) ([http://www.ncbi.nlm.nih.gov/geo)](http://www.ncbi.nlm.nih.gov/geo) under series accession number [GSE8401](https://www.ncbi.nlm.nih.gov/geo/query/acc.cgi?acc=GSE8401). A total of 83 samples, including 31 PM (primary melanoma) samples and 52 MM (metastatic melanoma) samples, were available in the microarray dataset. The probe files were downloaded for analysis markers in the progression of malignant melanoma on Box-plots represent Student’s t-test: **P* < 0.05; ***P* < 0.01; ****P* < 0.001; *****P* < 0.0001).

*Luciferase Assay*

Melanoma cells were seeded in a 24-well plate, and transient transfections were conducted the following day using 2 mL Lipofectamine (Gibco-BRL) and 0.3 mg of PG13-Luc, a p53-dependent firefly luciferase reporter gene in a 200 μL final volume. The pCMVbGal plasmid was cotransfected to control the variability of transfection efficiency in the reporter assays. The day after transfection, metformin, CRO15 or actinomycin was added to the medium. 24 h after treatment, cells were harvested in 50 μL of lysis buffer and soluble extracts assayed for luciferase and ß-galactosidase activities. All transfections were repeated several times using different plasmid preparations. Luciferase assays were conducted exactly as previously described (Rocchi et al., 2001).

*Flow Cytometry*

Cells were treated for 24 h with 5 μM CRO15, 5 μM PLX4032 or 1 μM staurosporine for 6 h. Cells were detached with Hqtase and resuspended in specific buffer. For Annexin V-DAPI analysis, cells were incubated in Annexin buffer (Hepes 2 M, NaCl 5 M, KCl 1 M, MgCl_2_ 2 M, CaCl_2_ 2 M and H_2_O + 0.1 μL DAPI/100 μL) for 15 mins at 4°C in the dark. For caspase-3 active cell staining, a FITC Active Caspase-3 Apoptosis Kit from BD Pharmigen (550480) was used with the recommended protocol.

*In Vivo Murine Cancer Model*

Animal experiments were carried out in accordance with the Declaration of Helsinki and were approved by a local ethical committee CIEPAL (Comite Institutionnel d‘Ethique Pour l’Animal de Laboratoire-Azur). Female immune-deficient BALB/c nu/nu (nude) mice were obtained at 5 weeks of age from Envigo Laboratory (Gannat, France). Mice were randomly allocated in the different experimental groups and inoculated subcutaneously with A375 or A375-resistant melanoma cells (1.0x10^6^ cells/mouse). After tumor apparition (± 4-5 days, 50 mm^3^), animals received intraperitoneal injection of Labrafil (Control), PLX4032 (0.7 mg/mouse/day) or CRO15 (0.7 mg/mouse/day) dissolved in Labrafil. The growth tumor curves were determined by measuring the tumor volume with a caliper and using the equation V=(L*W^2^)/2. The variance is similar between the different groups except for the CRO15 group in which mice were responded homogeneously. This experiment wasn’t conducted blind. At the end of the experiment, mice were euthanized by cervical dislocation, and tumors were taken for western blot and immunofluorescence experiments. TUNEL assay was performed using the *In Situ* Cell Death Detection Kit (Roche, Meylan, France).

**Supplemental figure legends**

**Supplemental Figure 1: CRO15 inhibits ATP and activates AMPK**

1. A375 cells were treated with 5 μM CRO15 for 6 h before cell lysates were analyzed with a malachite green phosphate kit.
2. WM793 and Mewo cells were treated with 5 μM CRO15 for the indicated times. Lysates were analyzed by western blotting with the indicated antibodies. One representative experiment of three is shown.

C. NHM (Normal Human Melanocytes) and NHF (Normal Human Fibroblasts) isolated from foreskins were treated with AICAR 1 mM for 24h or with 5 μM CRO15 for the indicated duration. Lysates were analyzed by western blotting with the indicated antibodies. One representative experiment of three is shown.

S79 = Serine79; T389 = Threonine389; T172 = Threonine172; S2448 = Serine2448.

**Supplemental Figure 2: CRO15 activates the** **DNA repair pathway**

A. A375 cells were treated with 5 μM CRO15 for the indicated times. Lysates were analyzed by western blotting with the indicated antibodies; p-P38 (Thr180/Tyr182); p-IKKβ (Ser180), p-IkBα (Ser32). One representative experiment of three is shown.

B. Bio-informatic analysis of MELK and CDK1 transcript expression in primary and metastasis melanomas.

C. Primary human fibroblasts, melanocytes and keratinocytes were treated 48 h with 5 μM CRO15 or MELK inhibitor OTS167 at the indicated concentrations. Cell viability was estimated by trypan blue staining.

D. Immunofluorescence images of A375-resistant melanoma cells treated with DMSO or 5 μM CRO15 for 6 h. Active CHK2 was labeled with antibody (red), and DNA was visualized with DAPI (blue). Integrated density was determined for each condition on Fiji. Data are given as the median ±Min to max value of three independent experiments performed in triplicate. *p < 0.05; **p < 0.01; ***p < 0.001.

E. Immunofluorescence images of A375-resistant melanoma cells treated with DMSO, 5 μM CRO15 for 6 h or 1 mM Doxorubycin for 24 h. γ-H2AX was labeled with antibody (red), and DNA was visualized with DAPI (blue). Integrated density was determined for each condition on Fiji. Data are given as the median ±Min to max value of three independent experiments performed in triplicate. *p < 0.05; **p < 0.01; ***p < 0.001.

**Supplemental Figure 3: CRO15 activates the** **p53 pathway**

1. SK-MEL-28 cells were infected with an adenovirus encoding a functional p53 protein for 24 h and then treated with 5 μM CRO15. After 24 h, cell viability was determined by the trypan blue exclusion method. The results are expressed as percentages of the control and data given as the means ±SEM of three independent experiments performed in triplicate. *p < 0.05 ; **p < 0.01 ; ***p < 0.001.
2. SK-MEL-28 cells were infected with an adenovirus encoding a functional p53 protein for 24 h and then treated with 5 μM CRO15 for the indicated times. Lysates were analyzed by western blotting with the indicated antibodies. One representative experiment of three is shown.

**Supplemental Figure 4: CRO15 induces autophagy and apoptosis**

1. Integrated density from the experiment presented in 5B was determined for each condition on Fiji. Data are geometric means with 95% CI of three independent experiments performed in triplicate. *p < 0.05 ; **p < 0.01 ; ***p < 0.001.
2. A375 cells were pretreated with pepstatin and E64d for 2 h and then treated with 5 μM CRO15 or 400 nM rapamycin. After 24 h, cell viability was determined by the trypan blue exclusion method.
3. Lysates from cells treated as in B were analyzed by western blotting with the indicated antibodies. One representative experiment of three is shown.
4. A375 cells were treated with 5 μM CRO15 for 24 h and prepared for electronic microscopy analysis.
5. A375 cells were transfected with siLC3 and/or treated with 20 μM Q-VD before treatment with 5 μM CRO15. After 24 h, cell viability was determined by the trypan blue exclusion method.
6. A375 cells were transfected with siLC3 and/or treated with 20 μM Q-VD and then treated with 5 μM CRO15. Lysates were analyzed by western blotting with the indicated antibodies. One representative experiment of three is shown.

For B and E, the results are expressed as percentages of the control, and data are means ±SEM of three independent experiments performed in triplicate. *p < 0.05 ; **p < 0.01 ; ***p < 0.001.

**Supplemental Figure 5: CRO15 induces autophagy and apoptosis in a mouse model**

1. Mice from 7E were weighed 3 times a week.
2. Immunofluorescence on xenografts on BRAF inhibitor-resistant tumor sections. LC3B (green) and active caspase 3 (red) were labeled with antibody, and apoptotic cells were visualized with TUNEL kit (green). DNA was visualized with DAPI (blue).

**Supplemental Figure 6: CRO15 induces cell death in other cancer types**

Indicated cancer cell lines were treated for 48 h with 5 μM CRO15. Cell viability was determined by the trypan blue exclusion method. The results are expressed as percentages of the control and data given as the means ±SEM of three independent experiments performed in triplicate. *p < 0.05; **p < 0.01; ***p < 0.001.
