# Supplementary figures and images for: Discovery of a new molecule inducing melanoma cell death: dual AMPK/MELK targeting for novel melanoma therapies
Source: Cell Death Dis. 2021 Jan 11;12(1):64. doi: 10.1038/s41419-020-03344-6 (PMC7801734; doi:10.1038/s41419-020-03344-6)

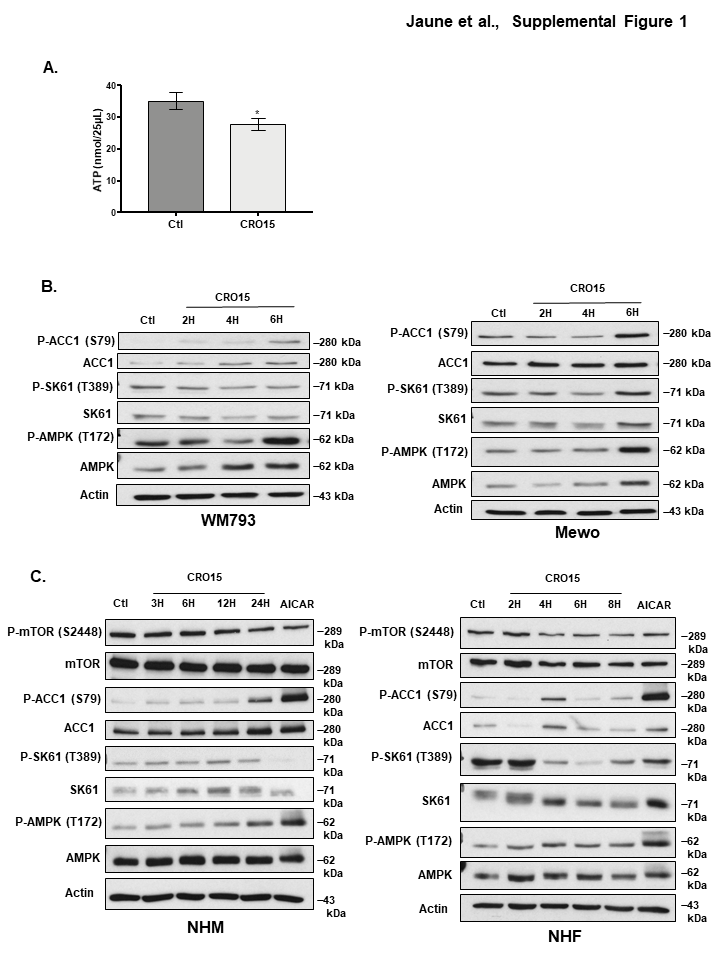

Supplement: Supplementary file 2 — Supplemental Figure 1 [file 41419_2020_3344_MOESM2_ESM.tif]

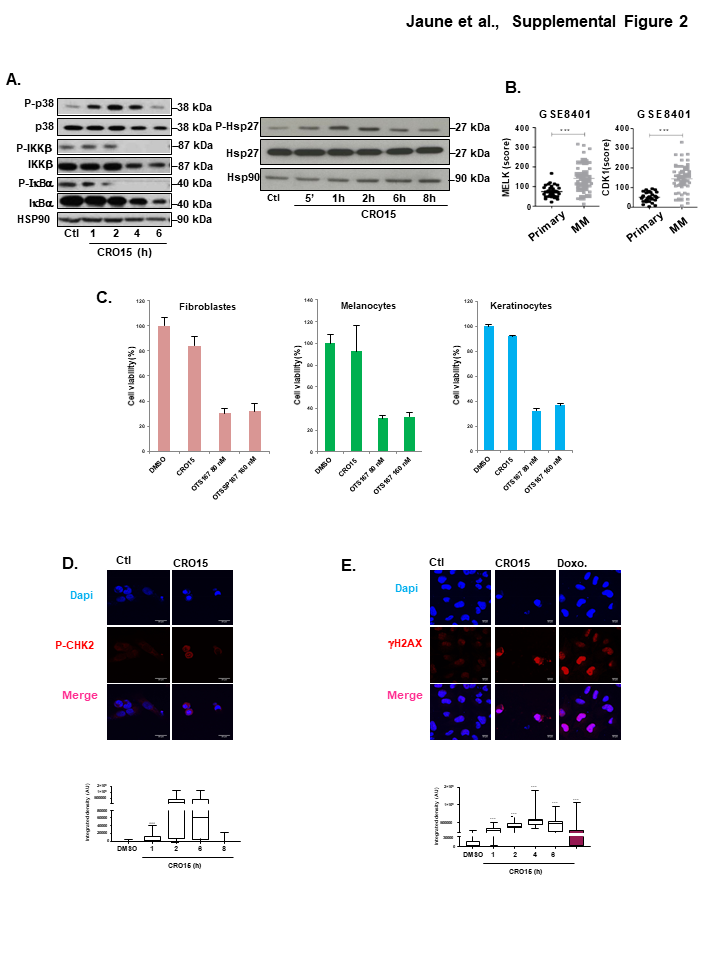

Supplement: Supplementary file 3 — Supplemental Figure 2 [file 41419_2020_3344_MOESM3_ESM.tif]

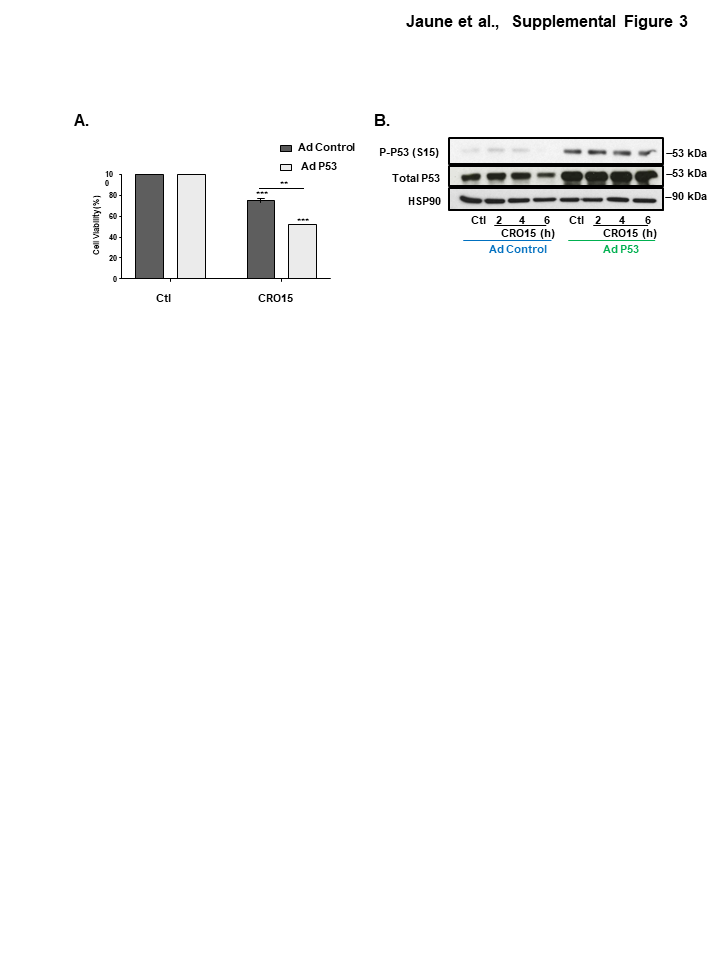

Supplement: Supplementary file 4 — Supplemental Figure 3 [file 41419_2020_3344_MOESM4_ESM.tif]

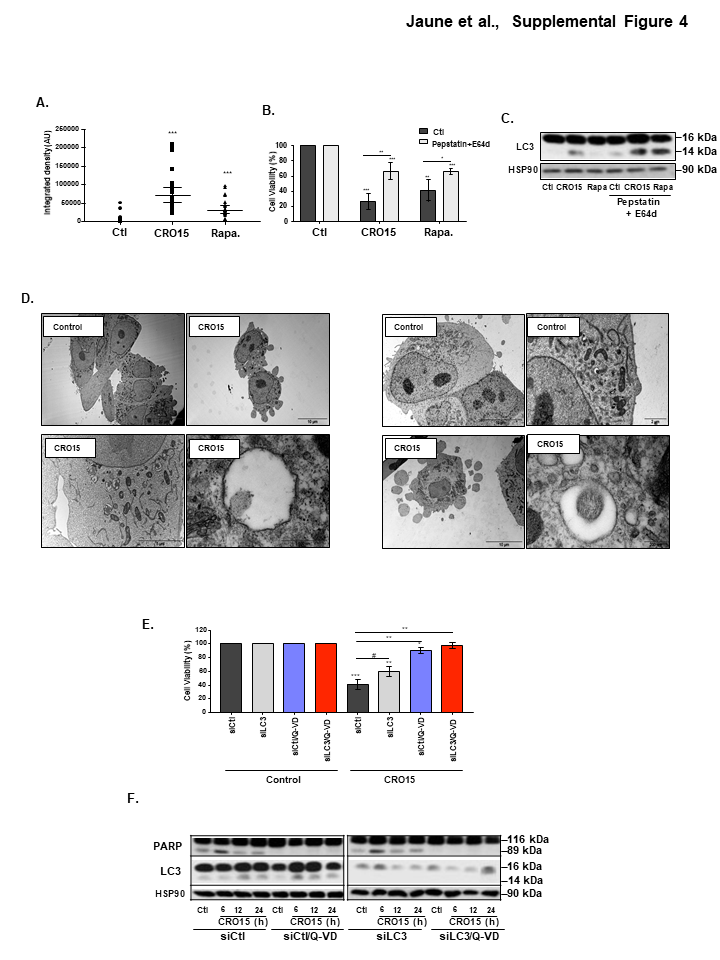

Supplement: Supplementary file 5 — Supplemental Figure 4 [file 41419_2020_3344_MOESM5_ESM.tif]

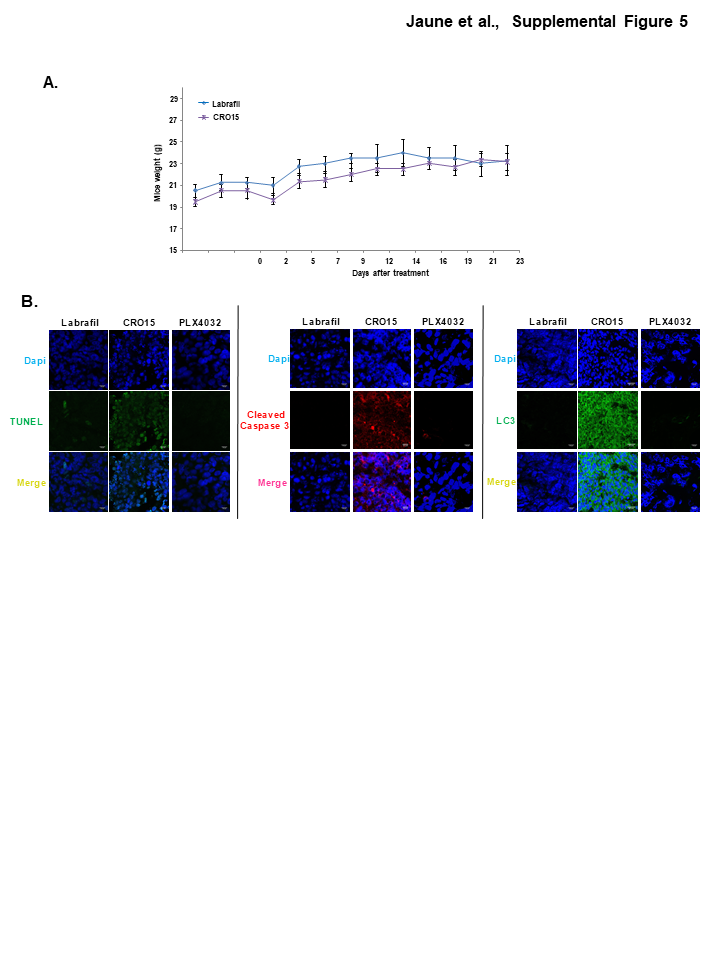

Supplement: Supplementary file 6 — Supplemental Figure 5 [file 41419_2020_3344_MOESM6_ESM.tif]

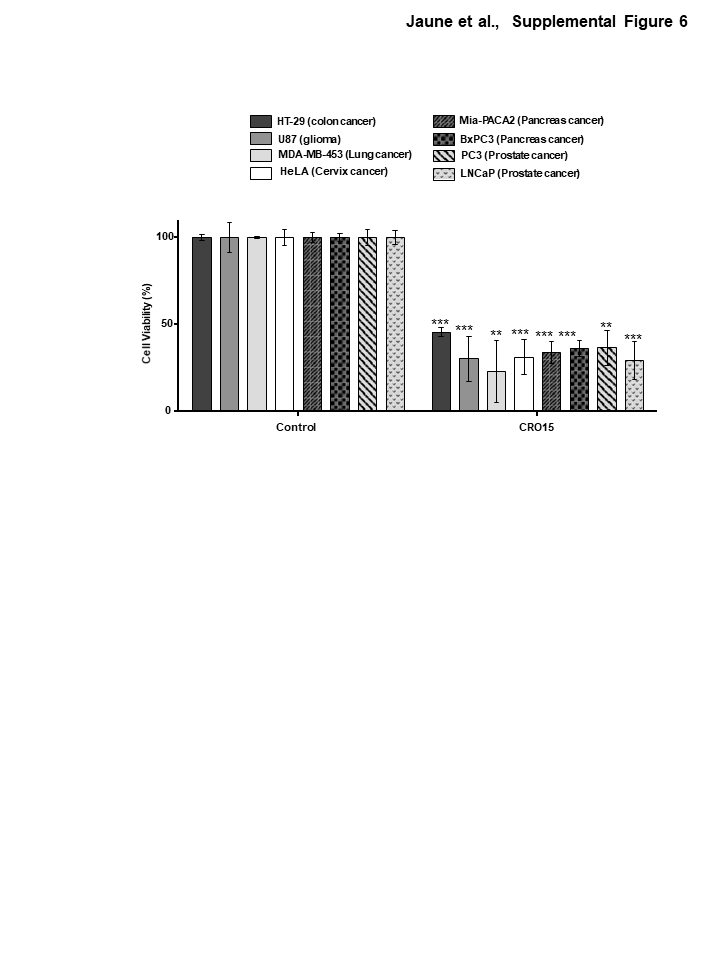

Supplement: Supplementary file 7 — Supplemental Figure 6 [file 41419_2020_3344_MOESM7_ESM.tif]
